# The Challenges in Developing Efficient and Robust Synthetic Homing Endonuclease Gene Drives

**DOI:** 10.3389/fbioe.2022.856981

**Published:** 2022-03-28

**Authors:** Sebald A. N. Verkuijl, Joshua X. D. Ang, Luke Alphey, Michael B. Bonsall, Michelle A. E. Anderson

**Affiliations:** ^1^ Arthropod Genetics, The Pirbright Institute, Pirbright, United Kingdom; ^2^ Department of Zoology, University of Oxford, Oxford, United Kingdom

**Keywords:** gene drive, gene editing (CRISPR-Cas9), DNA repair, germline, transgene expression, deposition, multiplexing

## Abstract

Making discrete and precise genetic changes to wild populations has been proposed as a means of addressing some of the world’s most pressing ecological and public health challenges caused by insect pests. Technologies that would allow this, such as synthetic gene drives, have been under development for many decades. Recently, a new generation of programmable nucleases has dramatically accelerated technological development. CRISPR-Cas9 has improved the efficiency of genetic engineering and has been used as the principal effector nuclease in different gene drive inheritance biasing mechanisms. Of these nuclease-based gene drives, homing endonuclease gene drives have been the subject of the bulk of research efforts (particularly in insects), with many different iterations having been developed upon similar core designs. We chart the history of homing gene drive development, highlighting the emergence of challenges such as unintended repair outcomes, “leaky” expression, and parental deposition. We conclude by discussing the progress made in developing strategies to increase the efficiency of homing endonuclease gene drives and mitigate or prevent unintended outcomes.

## 1 Introduction

Gene drive is the ability of a genetic element to bias its own inheritance. This allows gene drive elements to spread a genetic change through a population even while having a fitness disadvantage (“selfish-DNA”). Genetic engineering at scale through engineered/synthetic gene drives may allow many currently intractable public health challenges caused by pest species to be addressed. In particular, insect pests such as mosquitoes have life-history traits that may make them amenable to gene drive interventions (e.g., sexual reproduction and short generation times). The feasibility of using gene drives to fix a particular trait (population replacement) or suppress wild populations are both being investigated for addressing the harm caused by insect pests, in some cases with the same ultimate goal (e.g., eradication of malaria).

There are many examples of gene drives occurring in nature, acting through many different mechanisms (Burt and Trivers, 2006). Some types of gene drive rely on the action of sequence-specific DNA nucleases (enzymes that create DNA breaks). These have recently received a lot of attention by researchers following the discovery and characterisation of Clustered Regularly Interspaced Short Palindromic Repeats (CRISPR) systems (Jinek et al., 2012). The programmable CRISPR nucleases, of which CRISPR associated protein 9 (Cas9) is the most widely used, have provided researchers with powerful new tools to both facilitate genetic engineering and as constituent parts of gene drive mechanisms. Nonetheless, many important fundamental insights into building synthetic gene drive systems were gained before the use of CRISPR nucleases.

Double-stranded DNA breaks are a common occurrence in cells, and a range of mechanisms exist to resolve them. Under specific conditions, cells can use a homologous DNA template to prevent the loss of genetic information. This can be from an identical sister chromatid that is present during the S and G2 phases of the cell cycle, or the near identical homologous chromosome. Generally, in diploid organisms, each chromosome in a homologous pair is contributed by a different parent and contains the same content with minor sequence variation (sex chromosomes often are an exception). Therefore, interchromosomal repair within a homologous pair will result in loss of heterozygosity, but under most circumstances results in the genomic region retaining its function after repair.

**FIGURE 1:**
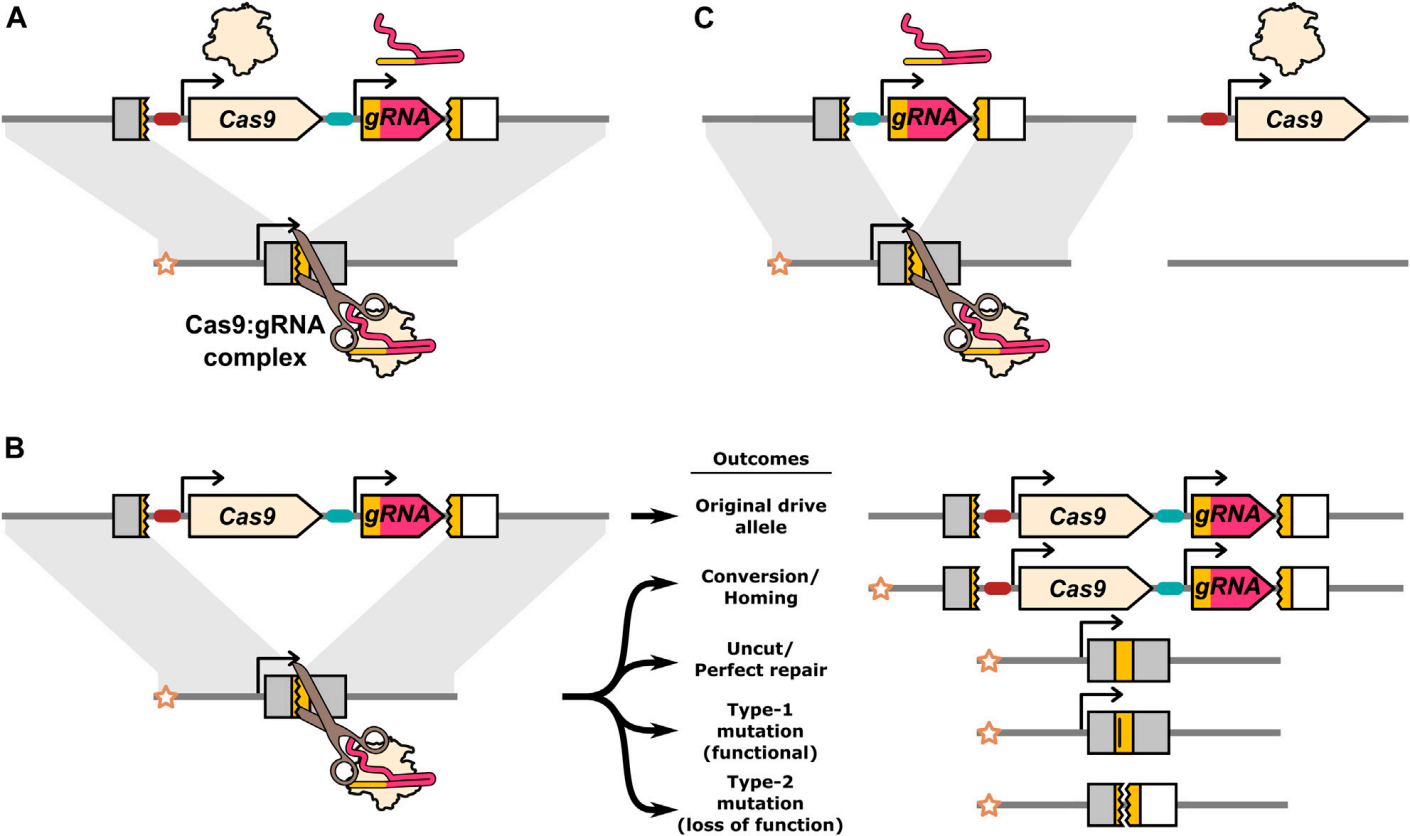
Illustration of the Cas9:*gRNA* homing endonuclease gene drive inheritance biasing mechanism and potential DNA repair outcomes. **(A)** The drive expresses Cas9 and a gRNA which together form a complex that find and cleave the target allele. **(B)** DNA breaks can be resolved by a range of different repair outcomes. Conversion occurs when HDR uses the homologous chromosome carrying the drive allele as a repair template. The star indicates the “recipient” chromosome, and allows the original drive allele to be distinguished from a drive allele produced by homing. Alleles that are cut and repaired perfectly, as well as uncut alleles, remain unconverted. In addition to conversion, DNA repair can also result in mutations in the target gene. If the specific mutations do not disrupt the function of the target gene they are classified as type-1. If the mutations do prevent normal function of the target gene they are classified as type-2. **(C)** A component essential to the inheritance biasing process such as the gRNA or Cas9 gene can be located on a separate element producing a “split-drive” configuration. The gRNA target sequence in the endogenous target gene is indicated by a yellow colouring. The target gene is shaded grey, unless disrupted by the drive or type-2 mutations at which point it is shaded white to indicated its loss of function. Type-1 mutations lose the gRNA target sequence (indicated by a vertical bar in the yellow target site), but remain functional.

Homing endonuclease gene drives (HEGs) can induce their own switch from a heterozygote to a homozygote state by creating a DNA break in the “recipient” homologous chromosome corresponding to the locus of the HEG genetic material on the “donor” homologous chromosome (Figure 1A). In effect, the coding sequence for the HEG may then be identified as missing from the cut chromosome and the HEG and linked sequences are copied over during repair of the DNA break (Figure 1B). If the transformed cell is part of the organism’s germline lineage, the gene drive element will be propagated to the next generation with a higher frequency than would be expected from normal Mendelian inheritance. This copying or “homing” process can repeat itself in subsequent generations and allows the HEG element to increase in frequency in a population, along with any associated genetic modifications that affect the desired change in the population.

In general, the HEG drives we describe here are designed and optimised for the homing inheritance bias mechanism. However, there are a number of ways through which nuclease-based drives have been described to bias their inheritance with seemingly subtle changes underlying the difference in mechanism. For almost all HEG studies, there is limited evidence on the actual underlying mechanism(s) giving rise to any observed inheritance bias and recent evidence suggests the mechanisms may be more heterogeneous than previously understood. An important hallmark of the homing process is the copying of the drive element onto the recipient chromosome. Many other nuclease drive mechanisms instead operate through decreasing the inheritance of the nondrive recipient chromosome. We will use the term inheritance bias or estimated homing when the specific experimental set-up was not strictly able to distinguish between inheritance bias through copying (homing) or exclusion of the chromosome not carrying the drive allele.

Synthetic HEGs have, in almost all cases, been inserted into and targeted the sequence of an endogenous gene or targeted a separately inserted synthetic target gene (e.g., GFP). A principal reason for this is DNA sequence constraints. Many simultaneous DNA breaks in the genome may result in DNA damage-induced cell stress (Aguirre et al., 2016) and chromosomal rearrangement (Kosicki et al., 2018). As such, synthetic HEGs are designed to only cut their specific target site and those targets are chosen to be unique within the genome. In addition, for HDR to occur, the region surrounding the DNA break must be (relatively) uniquely matched with the homologous chromosome, as homologous loci elsewhere in the genome may compete as evidenced by homing from non-paired sites (Chan et al., 2011; Lin and Potter, 2016). Lastly, for the drive to affect a significant proportion of a population, its target must also be present in most individuals of the target population. These sequence constraints are generally only found in the (coding sequence) of highly conserved genes.

Beyond the sequence constraint, there are additional benefits that may come from placing HEGs in an endogenous gene. The “effector” function of synthetic HEGs (e.g., female recessive sterility) may be most readily achieved by disrupting a specific endogenous gene directly with the drive element (Burt, 2003). In research contexts, the target is often a gene that provides a phenotypic readout when disrupted. In addition, the chromatin environment associated with an endogenous (expressed) gene may be more permissive to the expression of the inserted transgenes (O’Brien et al., 2018; Brady et al., 2020; Dhiman et al., 2020), and an endogenous gene’s promoter may even be directly used to express the drive genes (Nash et al., 2019; Weitzel et al., 2021). The target gene’s chromatin context may also influence Cas9 cutting efficiency and DNA repair (Verkuijl and Rots, 2019). Lastly, targeting highly conserved essential genes is one of the most important tools for addressing unintended repair outcomes which will be discussed more later.

Generally, the ability of a synthetic HEG to spread will depend on whether its efficiency at biasing its own inheritance can overcome its associated fitness costs. These costs depend on a number of factors: the particular application will matter, as population modification with a “neutral” modification such as insecticide susceptibility or pathogen resistance will likely impose a far lower fitness cost than a modification designed to suppress the target population (cause a population decline). The actions of the drive machinery itself will also apply some fitness cost, and characteristics of the target species and population, such as size, density, gene flow, and density dependence will all factor into the drive requirements. More complex HEG designs required for “self-limiting” drives (Noble et al., 2019) may also place higher requirements on the drive efficiency. Moreover, the HEG efficiency will also influence the required release frequencies, and the logistical costs and feasibility associated with the use of that particular system. As such, understanding and improving gene drive inheritance biasing efficiency and fidelity may allow for application in currently refractory species, and possibly decrease the cost of already feasible interventions.

To our knowledge, synthetic HEGs have, with varying inheritance biasing efficiencies (in some cases none), been reported in 9 species: *Saccharomyces cerevisiae*, *Candida albicans*, *Arabidopsis thaliana*, *Drosophila melanogaster*, *Plutella xylostella*, *Anopheles gambiae*, *Anopheles stephensi*, *Aedes aegypti*, and *Mus musculus*. The research field has learnt much about the factors influencing HEG outcomes and explored different strategies for optimisation. However, much remains unknown, such as what specifically constitutes an efficient HEG, and what underlies the different outcomes observed with different drive designs. We will first present an overview of the field and the milestones achieved in the development of synthetic HEGs so far. Then we will discuss in detail specific technical challenges in developing efficient HEGs and end with potential solutions.

## 2 Milestones in the Development of Synthetic HEGs

### 2.1 Transferring a Natural Homing Endonuclease Gene Drive

Natural HEGs were first identified in unicellular eukaryotes, fungi, and plants (Burt and Trivers, 2006) and it was initially unknown if HEG based inheritance biasing mechanisms would function in animals. *S. cerevisiae* has a natural gene drive that relies on the I-*Sce*I meganuclease, which in its endogenous context cuts the large rRNA sub-unit of the biparentally inherited mitochondria (Monteilhet et al., 1990). Chen et al. integrated the I-*Sce*I nuclease into a synthetic docking site in *D. melanogaster* and separately inserted a fluorescent protein into the docking site to function as a recipient chromosome (Chan et al., 2011). They for the first time demonstrated that a synthetic HEG could bias its own inheritance in animals. In that, and a follow-up study, they performed extensive tests of the I-*Sce*I drive with different regulatory sequences upstream (promoter and 5′UTR) and downstream (3′UTR) of the I-*Sce*I nuclease coding sequence (Chan et al., 2011, 2013a). The best performing drive used the promoter of the Rcd-1r gene and the *β*-Tub56D 3′UTR aiming for spermatogenesis-specific expression. This drive converted 23% of the target alleles located on the recipient chromosome, achieving an overall inheritance of ≈62% (≈50% from donor chromosome + ≈12% converted recipients). The majority (63%) of target alleles on the recipient chromosome appeared to remain unmodified, likely uncut.

While a substantial number of promoter/3′UTR combinations achieved higher cut rates than Rcd-1r/*β*-Tub56D, they resulted in lower or even no HEG conversion. Instead of copying the HEG element through HDR, alternative DNA repair pathways created mutations at the site of DNA cleavage. These results indicated that simply creating a DNA break was not enough for efficient homing, the timing of nuclease expression was seemingly essential for efficient conversion. In addition to potentially competing with the homing process, these mutations create cut-resistant alleles that are inherited by the next generation. Mathematical modelling and cage trials have indicated that these cut-resistant alleles can prevent a drive from reaching fixation, or even spreading effectively in real world applications (Marshall et al., 2017; Noble et al., 2017; Champer et al., 2018; Pham et al., 2019; Champer S. E. et al., 2021; Fuchs et al., 2021).

Work by Windbichler et al. demonstrated that the I-*Sce*I HEG could also function in the disease-relevant *A. gambiae* mosquito (Windbichler et al., 2011). Expressed from a male specific promoter, the HEG reached inheritance rates of 86%. Moreover, they showed using small scale cage experiments the first evidence that a synthetic HEG could spread within a receptive population. Despite similar, and many additional promoter/3′UTR combinations having been tested in *D. melanogaster*, the inheritance bias achieved with the *A. gambiae* drive was higher. This was the first suggestion that some organisms are more receptive to HEG based inheritance biasing than others. Yet, even in *A. gambiae* the inheritance bias was likely too low for most applications. Moreover, these HEGs would not function outside of a specifically modified lab strain: both *D. melanogaster* and *A. gambiae* do not naturally contain the I-*Sce*I target sequence and in each case, a synthetic target allele had to be created for the drives to function.

### 2.2 The Cost of Re-Targeting

To address the targeting limitations, Chan et al. used site-directed mutagenesis of the I-*Onu*I meganuclease to change its recognition sequence to allow it to target a closely related sequence naturally found in *Anopheles* mosquitoes (Chan et al., 2013b). They placed this *Anopheles* target in a GFP reporter and tested the inheritance bias in *D. melanogaster* males using the Rcd-1r promoter and *β*-Tub56D 3′UTR. While two I-*Onu*I variants biased their inheritance to the same degree as the I-*Sce*I drive (Chan et al., 2011), they only did this with far higher overall cut rates (therefore generating more mutations). Moreover, there were indications that a mutation introduced into the I-*Onu*I catalytic site to achieve these higher cut rates was causing reduced fertility, possibly due to sequence promiscuity. Furthermore, the *Anopheles* gene that the modified I-*Onu*I could target happened to closely match the natural I-*Onu*I targeting sequence and would not in itself confer disease resistance or allow population suppression. Clearly, the creation of synthetic HEGs would greatly benefit from more programmable, yet specific, nucleases.

The first and second generation of programmable nucleases came in the form of zinc-finger nucleases (ZFNs) and transcription activator-like effector nucleases (TALENs). The only reported use of ZFNs and TALENs in a HEG system was by Simoni et al. (Simoni et al., 2014) with the Rcd-1r promoter and *β*-Tub56D 3′UTR in *D. melanogaster*. In males, ZFN and TALEN-based HEGs achieved homing rates of 34% and 49%, respectively. These homing rates were higher than with I-*Sce*I, but with equivalent or worse cut-to-homing ratios. While the TALEN HEGs had overall higher homing rates and better cut-to-homing efficiency than the ZFN HEGs, only the ZFN HEGs were able to spread significantly within small cage trials. It became apparent that the programmability of these nucleases came at a cost: repetitive genetic sequences. ZFNs and particularly TALENs are composed of large repeating DNA binding “units” in which only a few amino acids are changed to specify the target sequence. This resulted in repetitive drive constructs, which in turn were found to make the drive unstable, losing function at a high rate due to internal recombination and/or partial homing. Only 40% of the TALEN and 75% of the ZFN inheriting progeny resulting from (partial) homing in the first generation could themselves home in the next generation.

Together, the above work demonstrated that the HEG mechanism could indeed work in animals and spread in small cage populations. However, the difficulty in programming meganucleases, and the shortcomings of ZFNs and TALENs meant that with the available tools it would be a momentous task to create an effective HEG drive system that would spread in non-ideal conditions. That is, until the discovery of the CRISPR nucleases.

### 2.3 Programmable RNA Guided Gene Drives

Starting in 2012, a new generation of molecular tools greatly accelerated our ability to perform gene editing. Central to this revolution has been the discovery of new easily programmable nucleases, the best-known version being Cas9 from the type-II CRISPR system of *Streptococcus pyogenes* (Jinek et al., 2012). Cas9 can, with few limitations, be targeted to almost any DNA sequence by straightforward RNA to DNA base pairing through a short “guide” RNA (gRNA). If a sufficient match is found (not necessarily perfect), Cas9 will then create a double-stranded break. In a very short time-frame CRISPR based synthetic HEGs were reported in *S. cerevisiae* (DiCarlo et al., 2015), *D. melanogaster* (Gantz and Bier, 2015), *A. stephensi* (Gantz et al., 2015), and *A. gambiae* (Hammond et al., 2016).

The first CRISPR HEG reported in yeast demonstrated near perfect ( > 99%) inheritance over multiple generations, and in multiple strains (DiCarlo et al., 2015). Moreover, this work demonstrated the feasibility of using HEGs with more advanced modifications which had been previously proposed (Burt, 2003; Esvelt et al., 2014). This included carrying a cut-resistant, but functional version of the target gene on the HEG allele, reversing the changes of one drive with another, and split-drives. In a split-drive, one component essential to the drive mechanism is housed on a separate locus, generally by separating Cas9 from its gRNA (Figure 1C). This allows the HEG that carries the gRNA to be safely tested, as it will only behave like a HEG in a lab strain that already expresses Cas9 and will not spread in wild populations. The synthetic target sites needed for earlier nucleases provided similar protection against unintended spread beyond the laboratory. The publication of CRISPR HEGs in yeast demonstrated that CRISPR gene drives are capable of extremely high conversion efficiencies and gave an initial indication that the CRISPR drives do not suffer from the same genetic instability issues seen with previous programmable nucleases. While these results were encouraging, the natural I-*Sce*I HEG also worked extremely well in yeast but failed to reach similar efficiencies in animals. Fortunately, the first CRISPR HEGs in *D. melanogaster* (Gantz and Bier, 2015), *A. stephensi* (Gantz et al., 2015), and *A. gambiae* (Hammond et al., 2016), each used a *vasa* regulatory element and reported inheritance rates over 90%, a massive improvement over the non-CRISPR HEGs. However, each publication also laid bare challenges that could prevent the effective spread of CRISPR HEGs.

Gantz et al. reported the first CRISPR HEG in *D. melanogaster* (Gantz and Bier, 2015). The HEG was inserted in and disrupted the X-linked *yellow* gene, limiting drive to XX females only. Gantz observed loss of function of the yellow gene target in almost all progeny (97%) of gene drive heterozygous mothers. This also occurred in female progeny suggesting that the maternally inherited drive converted the paternally contributed functional yellow allele in the early embryo. However, later publications found substantially lower inheritance rates (76–85%) with a near-identical constructs but including a fluorescent marker (Champer et al., 2017; Xu et al., 2020). It is probable that part of the seemingly super-Mendelian inheritance of the HEG observed in the earlier study (Gantz and Bier, 2015) was due to the maternal “deposition” of the Cas9:gRNA complex without inheritance of the drive expressing allele itself (Xu et al., 2020). The deposited nuclease and gRNA could cause the disruption of the *yellow* target gene in the absence of inheritance of the HEG itself. This could be problematic, as even with high rates of HDR the (repeated) cutting in individuals that did not inherit the drive allele will lead to mutations in the target gene. Moreover, the first HEG reported in *A. stephensi* showed that even when the drive is inherited to serve as an HDR template, deposition can have a negative effect.

Gantz and Jasinskiene et al. reported the development of a synthetic CRISPR HEG in *A. stephensi* (Gantz et al., 2015). Drive inheritance was scored separately in the progeny of males and females that inherited the drive allele from their father, and in the progeny of males and females that inherited the drive allele from their mother. Heterozygous parents of either sex passed along the drive element to 98–99% of their own progeny when that parent inherited the drive allele via the paternal line (drive carrying grandfather). However, strikingly, the maternal contribution of the drive allele (and accompanying maternal deposition) caused germline conversion rates to sharply drop, with only 56% of progeny from males and 62% of progeny from females having inherited the drive element. Moreover, unlike autonomous expression from the drive allele, the maternally deposited nuclease also affected somatic tissues. Maternal deposition was seemingly resulting in nuclease activity early in the embryo when HDR was not favoured, converting the target alleles to resistant alleles that could no longer be converted when expression occurs in the germline and HDR is favoured. Even if deposition-based conversion had been efficient, somatic drive activity has the possibility to cause its own issues. This was most strikingly highlighted by the first *A. gambiae* CRISPR HEG targeting candidate population suppression genes.

Hammond et al. identified three genes that, when disrupted, confer a recessive female sterility phenotype in *A. gambiae* (Hammond et al., 2016). They created Cas9 HEGs in each gene and demonstrated extremely high inheritance rates in both females and males (99%). However, the HEG heterozygous females unexpectedly produced only 0–9% of the number of larvae wild-type females did–a sterility effect that was intended to be limited to the drive homozygotes. They showed that *vasa*2-Cas9 expression was not fully germline restricted, and the nuclease was being expressed in somatic tissues. This lead to nuclease activity in some somatic cells, causing the remaining functional copy of the target gene to be lost, and the recessive phenotype to present in (initially) heterozygote individuals. Additionally, they identified mutations in the female fertility genes that, while preventing Cas9 cleavage, did not disrupt the normal function of the genes. They proposed that depending on the reproductive load of the drive, these functional resistant mutations could prevent the collapse of the target population and demonstrated this in a follow-up publication (Hammond et al., 2017).

Together, this initial set of studies demonstrated that maximising inheritance bias, while minimising unintended fitness costs and the creation of inheritable resistance mutations, remains a challenge with Cas9 based HEGs. Moreover, subsequent studies with HEGs in *M. musculus* (Grunwald et al., 2019; Pfitzner et al., 2020; Weitzel et al., 2021), *A. aegypti* (Li et al., 2020; Verkuijl et al., 2020; Reid et al., 2021), *P. xylostella* (Xu et al., 2021), and *Arabidopsis thaliana* (Zhang et al., 2021) have generally proved less efficient than in *Drosophila* and the *Anopheles* mosquitoes. Unintended DNA repair outcomes, inopportune drive expression, and deposition have emerged as the most important impediments for developing efficient HEGs.

## 3 The Main Technical Challenges Facing Synthetic HEGs

### 3.1 Unintended DNA Repair Outcomes

In most cases, the highest possible inheritance biasing rate is desired when developing a HEG. However, target alleles that remain uncut may be converted in the following generations, whereas cut-resistant mutations cannot. As such, the ratio between drive conversion events and unintended outcomes can often be more important than the inheritance biasing rate alone. HEGs may be more susceptible to resistance than other gene drives because DNA damage and repair are directly involved in their inheritance biasing mechanisms. This means a HEG can directly create resistance to itself that was not already present in the target population. This “induced” resistance is in the form of sequence changes to the target allele by unintended DNA repair pathways, often collectively described as non-homologous end joining repair (NHEJ), that prevent further cutting by the nuclease. Problematically, this resistance, if arising anywhere in the germline lineage, can, in addition to lowering the inheritance biasing efficiency of the drive, also be inherited and contribute a new resistant allele to the population’s gene pool.

The consequence of the mutation depends both on the target gene and the nature of the DNA lesion. With “type-1” resistance mutations preventing cutting by the nuclease, but otherwise leaving the function of the target gene intact, and ‘type-2” mutations preventing cutting by the nuclease as well as preventing regular function of the gene (Champer et al., 2017). Generally, type-1 resistance mutations are substitutions or small in-frame (n⋅3bp) insertions or deletions in the exons of protein coding genes. Type-2 resistance mutations are more likely with mutations causing a frame-shift in the exons of protein coding genes and large insertions and deletions. The importance of the amount and type of resistance mutations produced depends much on the particular application of the HEG. For a drive targeting a neutral locus, the distinction between type-1 and type-2 resistance will have no practical significance. In contrast, for a population suppression drive that aims to disrupt a particular (essential) gene, the ratio of type-1 and type-2 resistance alleles produced can be far more important than the overall amount of resistance alleles. Type-2 resistance alleles may slow the spread of the HEG, but they ultimately still contribute to the HEG effector function (disruption of the target gene). In contrast, even extremely rare type-1 alleles may allow for a population to quickly rebound or even be largely unaffected by a suppression HEG (Hammond et al., 2017).

**FIGURE 2:**
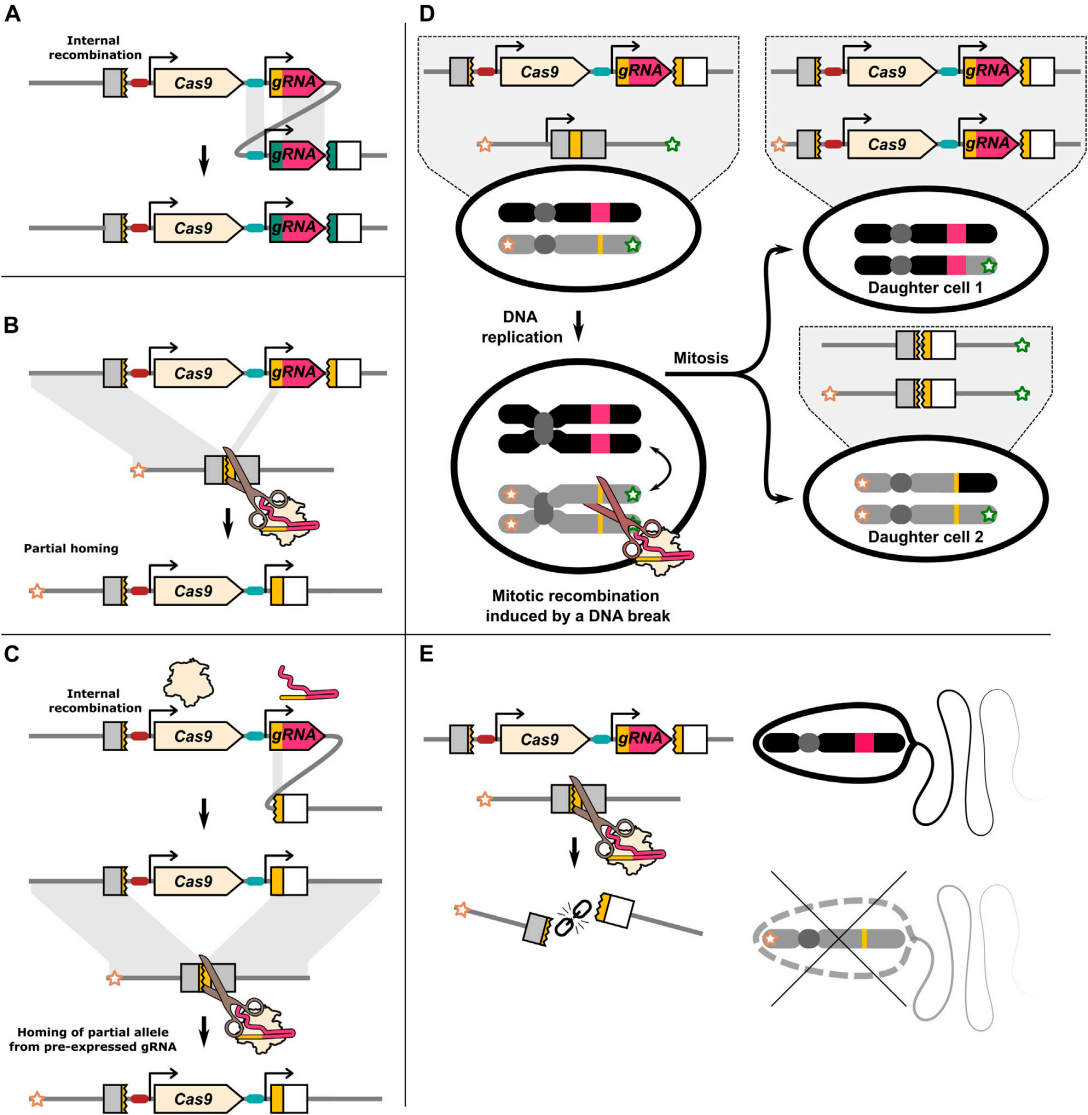
Illustration of DNA repair outcomes that may be associated with a HEG. **(A)** Repetitive sequences within the HEG may lead to internal recombination. The gRNA promoter and the constant gRNA “backbone” sequence in multiplexing drives may be particularly susceptible to internal recombination. **(B)** Partial homing by means of the gRNA target sequence located on the drive allele. The gRNA gene contains sequence homology to its target. This may allow for partial homing. **(C)** Recombination of the gRNA target sequence with a partial target sequence adjacent to the drive element. If the resulting allele homes it may resemble the product of partial homing even if a chromosome marker is present. **(D)** Cas9 induced DNA breaks have been reported to lead to mitotic recombination. This can produce two daughter cells that have loss of heterozygosity. Daughter cells generated by mitotic recombination can under some circumstances resemble products of homing. **(E)** In some cases a DNA break can lead to the loss of the target/recipient chromosome. This can result in inheritance bias through meiotic drive.

In addition to non-HDR outcomes, incomplete or internal HDR may also be a significant source of mutations. Alleles carrying parts, but not the whole drive element, have been reported in a number of publications studying CRISPR-Cas9 HEGs (Champer et al., 2017; Carrami et al., 2018; Champer et al., 2018; Oberhofer et al., 2018; Pham et al., 2019). In some cases, this can be explained due to internal recombination similar to what occurred with the repetitive ZFN and TALEN HEGs discussed earlier (Simoni et al., 2014). Oberhofer et al. tested a HEG with four gRNAs and found that the repetitive sequences introduced with multiple gRNAs likely caused the drive construct to internally recombine (Oberhofer et al., 2018) (Figure 2A). It is not clear if internal recombination is fully independent of the homing mechanism or if a significant fraction of the incomplete drive alleles are created through partial HDR from an otherwise intact donor allele. In some cases, the recovered incomplete drive alleles more strongly suggest they emerged due to partial copying.

In contrast to I-*Sce*I, ZFN or TALENs, Cas9 identifies its target through Watson-Crick base pairing of ≈20 nucleotides of the gRNA with the genomic DNA. Some publications have specifically identified partial drive alleles that are consistent with the gRNA gene target sequence having been used as one of the homology ‘arms’ during the homing process (Pham et al., 2019; Champer et al., 2017). This results in only part of the drive allele being identified as “missing” from the recipient chromosome, generating a partial copy (Figure 2B). However, different repair processes may give the same ultimate product (Figure 2C). Internal recombination and partial homing may be more common than is currently recognised. In almost all cases, inheritance rates are determined by scoring the presence of a dominant fluorescent gene linked to the HEG which may also be a partial drive allele (Oberhofer et al., 2018). While in most cases incomplete HDR should result in a type-2 resistance mutation, these partial homing events may pose a problem for drives with distinct cargo genes or sequence changes. Of particular importance, HEGs have been developed that carry sequences that rescue the function of the gene that they disrupt and partial homing of only these rescue sequences may create type-1 resistance alleles.

There may be a set of DNA repair outcomes, such as mitotic recombination and meiotic drive, that are underappreciated because they do not leave a distinct mutational signature. With mitotic recombination, DNA repair causes a dividing cell to produce daughter cells, where one daughter cell has two copies of a paternal chromosome region, and the other has two copies of the maternal chromosome region (Figure 2D). The production of individual cells homozygous for a particular parental gene resembles the outcome of homing, however, mitotic recombination does not directly bias the inheritance of any allele as reciprocal cells homozygous for the other allele are also created. However, in one study, *D. melanogaster* females carrying a single copy of the dominant female sterility inducing ovo^*D*1^ transgene could nonetheless produce viable offspring due to mitotic recombination induced by nos-Cas9 (Allen et al., 2021). Mitotic recombination can seemingly be a substantial outcome of DNA damage, and may be relevant in a HEG with a dominant acting effector such as sex-conversion. Mitotic recombination is most commonly studied by targeting both homologous chromosomes (Brunner et al., 2019; Allen et al., 2021), however it has been demonstrated to occur when only one homolog can be cut (Sadhu et al., 2016).

Finally, if DNA repair fails altogether, the cut recipient chromosome may instead be lost (Figure 2E). Loss of haploid cells or fertilised embryos carrying the recipient chromosome will in effect increase the relative inheritance of the donor chromosome, providing a potential separate mechanism of inheritance bias for HEG drives. If the recipient chromosome is marked, inheritance bias through homing or through the loss of the recipient chromosome can be distinguished. An under representation of the recipient chromosome marker has been reported in multiple publications with an element otherwise expected to function through homing (Guichard et al., 2019; Xu et al., 2020; Terradas et al., 2021b), with in some cases meiotic drive seemingly exclusively mediating the observed inheritance bias (Li et al., 2020; Verkuijl et al., 2020). In one study, under representation of a restriction enzyme site nearby the I-*Sce*I cut site on the recipient chromosome was suggested to be due to DNA repair after homing also replacing the nearby marker (termed “co-conversion” or “copy-grafting”) (Windbichler et al., 2011). All the pre-CRISPR HEG studies we discussed preformed crosses with marked chromosomes. However, for CRISPR HEGs only a small minority of studies have used a marked recipient chromosome, making it difficult to judge the extent of this phenomenon.

Maximising the efficiency of HDR after a Cas9 induced DNA break is a major topic of research because of its broad applicability to biological and medical sciences (Nambiar et al., 2022). Most research into site-specific HDR has been with an exogenously supplied repair template, and less is known if or what the specific dependencies are for efficient interchromosomal HDR. In addition, many of the interventions that may be used to boost HDR may not be suitable for a gene drive context. Below we will discuss some specific alterations to HEG design that have been investigated in an attempt to steer the number of resistance alleles, the ratio between type-1 and type-2 alleles, and mitigate their effect once they do emerge. The most important of these has been to limit HEG expression to when HDR is more likely, which has a number of additional benefits. However, actually limiting drive activity to this “ideal” window has been challenging.

### 3.2 Spatial and Temporal Restricted Drive Expression

**FIGURE 3:**
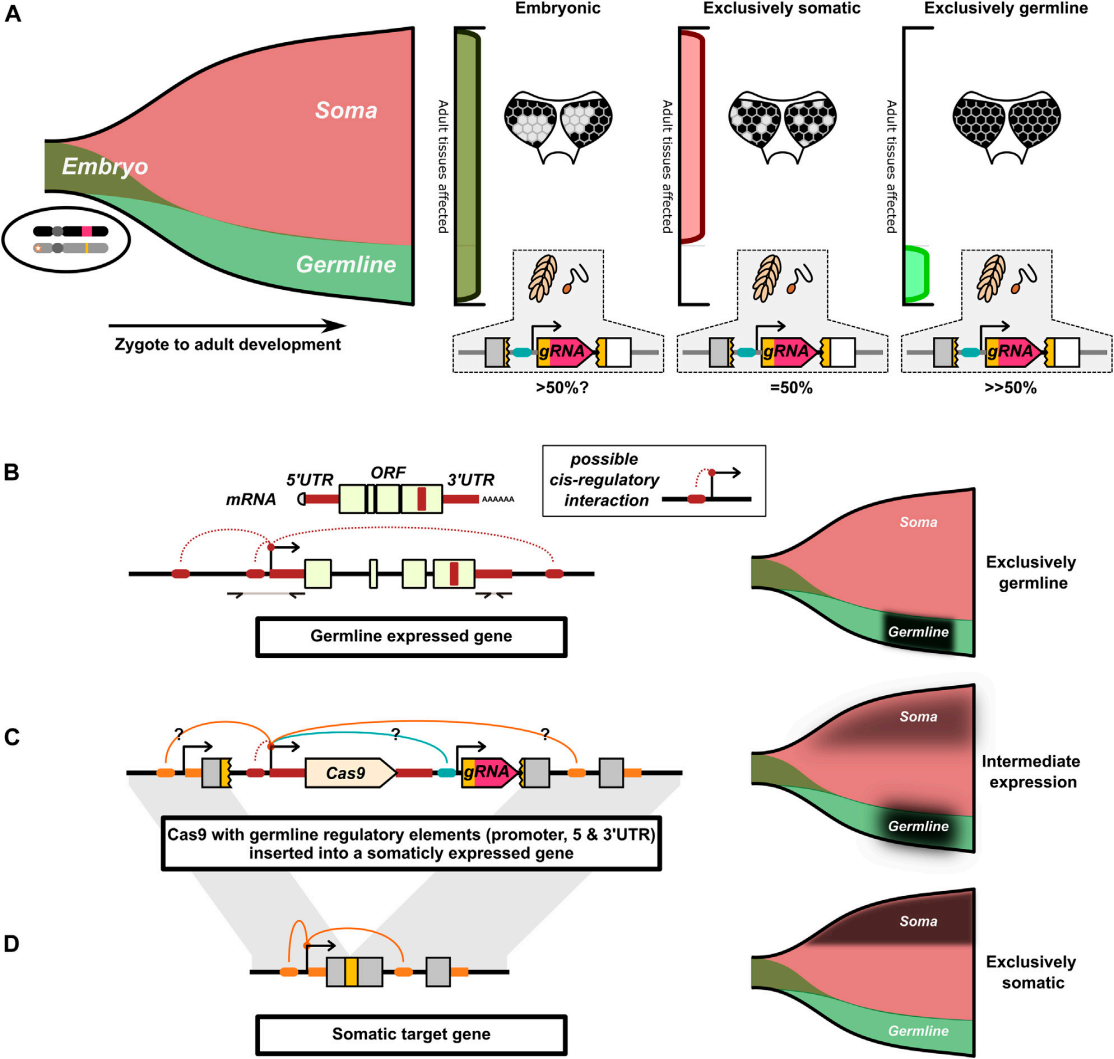
Illustration of the interaction of spatial and temporal restricted drive expression. **(A)** Drive activity in the early embryo can simultaneously affect cells of the somatic and germline lineages. Drive activity later in development can independently affect the germline and somatic cells. The funnel represents the cells that compose an individual as it develops from a zygote to an adult (left-to-right). Early in development, cells are simultaneously part of both the germline and somatic cell lineages. Only later in development do the germline and somatic cell lineages diverge. The pattern of somatic mosaicism in adult tissue can under certain circumstances be indicative of the timing of cutting, here indicated by different patterns of loss of pigment in the eye. In these cases, the later in development the somatic gene is disrupted, the more fine-grained the mosaic pattern. **(B)** Cas9 can be engineered to mimic the expression of endogenous germline restricted genes. This is commonly approached by use of the promoter, 5′and 3′ UTR of a germline restricted gene. The 5′ and 3′UTR can be identified from mRNA transcripts. Generally, a few Kb of sequence upstream of the 5′UTR are taken to capture the putative promoter. We have indicated additional speculative cis-regulatory interactions important for germline restricted expression that are not captured by this approach. **(C)** The Cas9 gene with the germline regulatory elements is inserted into the target gene’s locus. Cas9’s expression may be affected by cis-regulatory interactions with the target gene and with other drive components. We speculate that this may result in the Cas9 gene taking on an intermediate expression pattern resulting from the combination of different cis-regulatory interactions. **(D)** The target gene commonly has a somatic expression pattern that may not be conducive to homing. For (B–D), the proposed expression pattern of the respective allele is indicated by a black overlay on the funnels.

For a drive to function as a mechanism for super Mendelian inheritance, homing need only occur in the relatively small number of cells that make up the germline lineage (Figure 3A). Editing in any other cell lineage forming somatic tissues does not contribute to the drive inheritance biasing rate and in most cases not its effector function. Indeed, “somatic cutting” can be a significant source of additional fitness costs. In the most direct sense, many proposed population suppression drives will perform best when homing is tissue-restricted. These drives rely on heterozygote individuals being unaffected by a particular modification (e.g., disruption of a haplosufficent essential gene), yet passing the drive along at increased rates. This is achieved by restricting homing (and therefore induced homozygosity) to the germline. The effector modification remains heterozygous in tissues where it is required for normal function. An example of this is the *doublesex* targeting *A. gambiae* drive (Kyrou et al., 2018). More generally, for any drive, the unnecessary activity of the drive in somatic cell lineages may contribute to an additional fitness cost of the drive such as from off-target effects (Langmüller et al., 2021). This is compounded by the fact that the ability to perform HDR varies strongly by cell type and on-target resistance mutations that carry a significant fitness cost may be more likely to emerge in cells of somatic lineages.

A prominent hypothesis is that interchromosomal HDR after a DNA break is most likely if the DNA break coincides with meiosis I (Burt and Trivers, 2006; Xu et al., 2017; Champer et al., 2018, Champer et al., 2020 S. E.; Grunwald et al., 2019; Pfitzner et al., 2020; Terradas et al., 2021b; Terradas et al., 2021c; Kandul et al., 2021; Li et al., 2021; Taxiarchi et al., 2021; Weitzel et al., 2021; Xu et al., 2021). During this time chromosomal homologs exchange information through crossing-over events. The alignment of the homologs in the cell and activation of particular DNA repair machinery (Kadyk and Hartwell, 1992; Haber, 2015; Enguita-Marruedo et al., 2019) may make this timing more suited for interchromosomal copying of the HEG. As such, almost all synthetic HEGs have been designed to be active in the germline by flanking the nuclease transgene with the putative promoter, 5′UTR, and 3′UTR sequences of an endogenous germline specific gene (Figure 3B). The most widely tested have been sequences from the *nanos* and *vasa* genes.

There are a number of examples where the locus from which the HEG components are expressed seems to affect otherwise identical drive elements (Chan et al., 2013a; López Del Amo et al., 2020a; Grunwald et al., 2019; Reid et al., 2021), (Champer et al., 2019a) compared to (Champer et al., 2017; Champer et al., 2020 S. E.). One potential major challenge of achieving restricted expression is that a HEG inserted into an endogenous gene may be influenced by that gene’s cis-regulatory elements and broader chromatin context (Figures 3C,D), which under normal circumstances facilitate the specific expression pattern of the target gene. Ironically, for drives inserted into essential genes these regulatory elements may prime the HEG to express in the cells that drive activity would be most undesirable. This in effect, can make that locus one of the worst possible places for the HEG to be inserted to prevent “leaky” expression coinciding and interfering with the target gene’s activity. Split-drives, where the Cas9 is expressed at an unrelated locus, can avoid this regulatory element mismatch, but this may also cause them to be behave differently if reconstituted to a single element drive (Terradas et al., 2021a). The challenge of avoiding leaky expression is further compounded by some enhancers and other regulatory elements being located in the coding region of genes (Birnbaum et al., 2012). The presence of such elements in the target gene, and the absence of those elements from the germline genes, could alter the expression of Cas9 away from the germline restricted expression pattern the HEG is trying to recapitulate. Finally, the regulatory components of other drive components (e.g., fluorescent marker or cargo genes) may also interfere with the intended expression pattern of the nuclease and gRNA genes (Champer et al., 2019a).

Somatic cutting, in the absence of maternal deposition, has been reported for many Cas9 expression regulatory elements (Gantz et al., 2015; Champer et al., 2018; Kandul et al., 2020; Li et al., 2020; Verkuijl et al., 2020). As described above, this is generally detrimental, however, there are a limited set of cases where somatic conversion can be an intended part of the drive effector mechanism. Carrami et al. aimed to develop a sex-conversion drive disrupting the autosomal transformer (*tra*) gene (Carrami et al., 2018). By deliberately selecting promoters that would be active in somatic tissues, homozygous disruption of *tra* by the drive and somatic mutations would, in the medfly, convert XX females into fertile males (Pane et al., 2002). They performed their experiments in *D melanogaster* in which *tra* disruption leads XX individuals to develop into infertile pseudomales. However, in practice the XX individuals displayed an intermediate intersex phenotype (and were infertile). Males were unaffected by the somatic disruption of *tra* and displayed modest estimated homing rates (≈56%) in their germline. This work highlights that even in cases where somatic activity is desired, achieving a uniform disruption of the target gene in all cells can be a challenge. The outcome of such intermediate conversion, with some cells converted and others not, is called mosaicism and in many reports this is how somatic HEG activity presents.

While theoretically somatic and germline activity can be fully distinct processes, in some cases, the drive activity that gave rise to the somatic conversion did not necessarily (only) happen in the wrong cell lineages, but also at the wrong developmental time. Drive activity in the early embryo can convert cells that go on to give rise to both the germline and somatic tissue, producing the associated somatic phenotype later in development. In these cases, preventing leaky expression early in development may simultaneously lower the production of resistance alleles (if HDR is indeed not favoured) and decrease disruption of the target gene in somatic tissues.

Another potential source of resistance mutations occurs at the other end of differentiation, post meiosis. Any recipient chromosomes that escaped cutting in the germline will be separated from the donor chromosome once meiosis has occurred. If transcribed or translated drive components persist, cutting may occur after this point, resulting in repair by interchromosomal HDR being impossible. DNA damage near or post-meiosis may therefore be a source for resistance alleles (Champer et al., 2017; 2019a).

It is currently not clear if significant cutting occurs after meiosis. However, that expressed/translated HEG components persist into haploid cells that do not contain the HEG genes has been established for many drives. This is because the HEG components can go on to affect the fertilised zygote. This is the phenomenon of deposition we introduced earlier, and it has similar consequences to that of early embryonic leaky expression. However, there are important differences between the two processes.

### 3.3 Parental Effects (Deposition)

Many publications studying HEGs have noted that genetically identical individuals will show different somatic phenotypes and inheritance biasing efficiencies depending on which parent contributed the Cas9 and gRNA genes (Champer et al., 2017, 2018, 2019a; Carrami et al., 2018; Oberhofer et al., 2018; Guichard et al., 2019). These types of parental effects have even been observed in individuals that did not inherit any genetic components of the drive, indicating the deposition of already expressed drive components. In almost all cases, an exclusive maternal effect is observed, where a female carrying the HEG transgene(s) is thought to contribute the gRNA and/or nuclease protein/mRNA to her haploid eggs. While these parental effects are commonly referred to as deposition, it is important to note that for some HEGs an alternative or additional mechanism such as imprinting has not necessarily been excluded.

A key difference between “leaky” embryonic expression and embryonic cutting by deposition is that in the case of deposition, cutting can occur in the absence of inheritance of the drive. As such, even if interchromosomal HDR were favoured, deposition may result in the target allele being cut when not paired with a HEG allele. In addition, the activity of the deposited drive components may be expected to be early in development, affecting both the somatic and germline cell lineages. Cas9 protein half-life in cells and embryos has been estimated to be (substantially) less than 24 h (Kim et al., 2014; Burger et al., 2016). In *Drosophila*, the first meiotic divisions occur in third instar larvae ( > 2–days) in males, and early pupal stages ( > 3–days) in females (Hartenstein, 1993). This suggests that if Cas9 protein stability is limited to hours, it would not persist long enough to overlap with meiosis I in many species. Moreover, even if Cas9 protein persists to when meiotic divisions occur, most target alleles may already have been cleaved earlier in development. The activity window of deposited Cas9 mRNA is harder to predict. The mRNA’s translation in the embryo would likely delay and extend Cas9’s window of activity, while 5′ and 3′UTR sequences in the Cas9 mRNA copied from a germline gene may specifically limit the timing and location of translation. Moreover, it is possible that if mRNA deposition were to occur, the translated Cas9 would only become active in progeny that inherited the gRNA expressing gene, as the gRNA is highly unstable when not in complex with Cas9 protein (Hendel et al., 2015; Ma et al., 2016; Wang et al., 2019).

In general, deposition can be a substantial issue for effective homing, but it should be noted that the fitness costs associated with deposition-induced drive activity may be less detrimental than that of equivalent leaky expression drive activity (Beaghton et al., 2019). This is because deposition affects the progeny independently of if they have inherited the drive or not. In contrast, the fitness cost of leaky expression is limited to those individuals carrying the drive element. However, the possibility of creating inheritable (type-1) resistance mutations in non-drive inheriting progeny may still make deposition substantially more problematic.

Isolating and quantifying the resistance allele contribution of deposition can be difficult. Maternal deposition is most readily identified by disruption of the paternally contributed allele in the absence of inheritance of the drive allele. However, the maternally contributed allele may have been disrupted at the same time, or instead at any point in the mother’s development. As such, it is not possible to directly distinguish between germline resistance mutations and those that arise in the early embryo due to deposition; but there are two key pieces of evidence that may suggest a particular timing.

Firstly, particular DNA lesions have been found repeatedly in multiple offspring from the same parent (Champer et al., 2017, Champer et al., 2018; López Del Amo et al., 2020a). This can suggest that the specific mutation arose in the HEG drive parent’s germline, was replicated by cell divisions, and was passed along to multiple offspring. Interestingly, the fraction of offspring inheriting the same mutation may provide evidence for when it occurred in the parents germline (López Del Amo et al., 2020a). However, it should be noted that while NHEJ mutations are variable, their scope can be heavily influenced by the sequence context of the DNA break increasing the likelihood of identical mutations arise independently (Allen et al., 2019; Chakrabarti et al., 2019). Nonetheless, statistical analyses can detect if particular mutations co-occurred more in progeny of the same parent than between individuals with different parents (Champer et al., 2018).

The second observation is mosaicism. If there is a mix of mutant alleles found within an individual progeny, this indicates they arose after the first genome replication and did not arise in the parent’s germline. However, this analysis only works if the experiment is performed in such a way that the paternally contributed allele is cut-resistant and cannot in itself generate a mosaic outcome. As a caveat to this, Chan et al. has suggested that a mosaic phenotype observed in progeny that did not inherit their I-*Sce*I HEG allele was due to the inheritance and replication of an unrepaired DNA break instead of parental deposition (Chan et al., 2011). However, this was not further investigated.

Champer et al. reported that for a single drive element (comprising both *nanos*-Cas9 and a gRNA), the degree of embryonic cutting they saw in progeny subjected to maternal deposition and inheriting the drive was similar in the cases where the mother was heterozygous or homozygous for the drive (Champer et al., 2017). They offered that this “implies that most maternal Cas9 persisting to the [progeny’s] embryo stage was expressed after drive conversion events” in the mother. This was further supported by evidence that the rate of embryonic cleavage from deposition was lower when drive conversion was less likely, such as when mothers carried mostly resistance alleles instead of an additional wild-type target allele. Interestingly, in the progeny from these crosses the fraction of progeny with a somatic phenotype was similar regardless of whether a drive allele was inherited or not. This implied that most Cas9 persisted through to the embryo after maternal expression in diploid cells, rather than being expressed after meiosis and correlating with drive inheritance. These experiments provide some insight into the complex ways in which deposition can manifest for a particular expression pattern. In addition, it should be noted that deposition, or its interpretation, can be context dependent, as is well illustrated by the *zpg*-Cas9 *A. gambiae* drive. Crosses with maternal (Fuchs et al., 2021), paternal (Kyrou et al., 2018), or no deposition (Hammond et al., 2021a) have all been described for this same drive. Moreover, in *Drosophila* it has been reported that the embryo resistance rates can be strongly affected by the background genetics of crossed individuals (Champer et al., 2019b).

**FIGURE 4:**
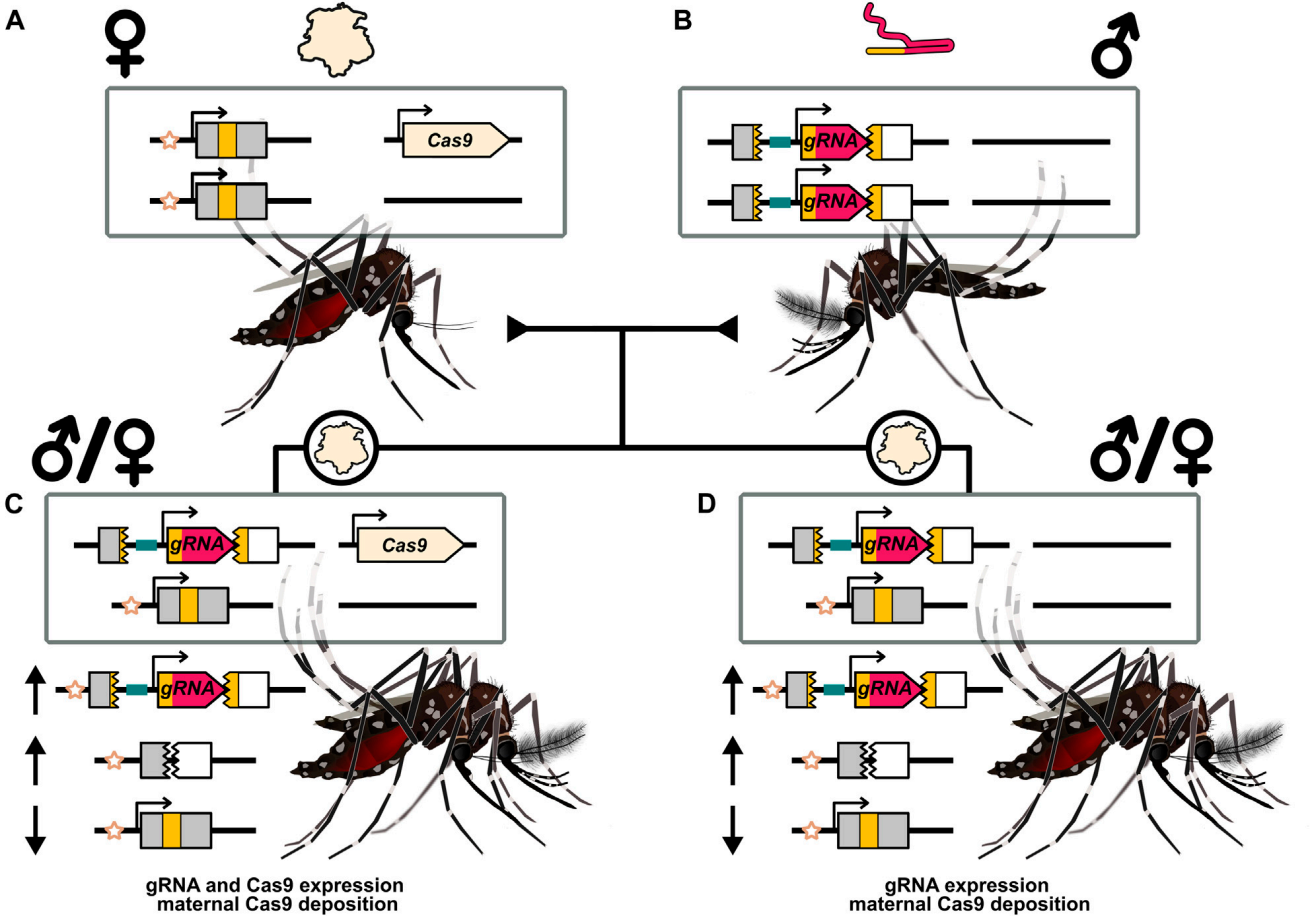
Illustration of maternal deposition and shadow drive in the context of a split-drive. **(A)** A female carrying a Cas9 split-drive element and depositing Cas9 protein into her eggs. **(B)** A male that carries the gRNA drive element and does not deposit the expressed gRNA. **(C)** Progeny of either sex that inherited the two transgenes from each parent separately. Expression of the Cas9 and gRNA genes can allow for inheritance bias. **(D)** Progeny of either sex that inherited the gRNA gene from their father but not the Cas9 gene carried by their mother. Maternal deposition can nonetheless provide a source of Cas9 that can complex with the expressed gRNA and mediate inheritance bias of the gRNA drive element. This is termed shadow drive. The recipient chromosome marker (star symbol) is used to indicate the target alleles that have been replaced by the drive element. Inheritance biasing mechanism of drive expression and/or deposition could operate through a non-copying mechanism. The arrows indicate a relative increase (upward arrow) or decrease (downward arrow) of the particular allele. While mosquitoes are illustrated Ramírez (2019a,b), these results have primarily been documented in *Drosophila*.

As introduced earlier, early embryonic nuclease activity associated with maternal deposition can cause a drive to affect somatic tissues (with the accompanying issues described above). While DNA repair associated with deposition-based cutting seems to be more error-prone, interchromosomal HDR does occur. The use of split-drives has allowed deposition to be investigated in more detail. Many HEGs have been tested in the form of a split-drive, where the Cas9 is located at an unlinked locus. Generally, this results in 50% of progeny inheriting the Cas9 gene independent of inheritance of the main drive allele carrying the gRNA(s). A number of publications have noted that individuals that only inherit the gRNA element can nonetheless pass it along at increased rates and this has been termed “shadow drive” (homing through deposited factors) (Champer et al., 2019a; Guichard et al., 2019; Kandul et al., 2020; Terradas et al., 2021b) (Figure 4). The activity window of deposited nuclease may be expected to be early in development with limited persistence. As such, examples of efficient shadow drive may provide a counter point to the hypothesis that homing is limited to meiotic cells that emerge later in development. Important in this interpretation is that deposition patterns can seemingly differ for the nuclease, gRNA, and Cas9:gRNA complex.

Kandul et al. created a split-drive in *D. melanogaster* targeting the *white* gene, and carrying a gRNA targeting the *yellow* gene in trans (Kandul et al., 2019). They tested four regulatory elements expressing Cas9 from a separate locus, and mediated significant inheritance bias with an average estimated homing rate of 73%. Interestingly, when the nuclease was carried by the grandmother, the estimated homing rate in the parent’s germline was roughly the same (69%), even when the Cas9 gene had not been inherited. Shadow drive through maternal deposition with the four Cas9 regulatory elements they tested was seemingly just as efficient at biasing the inheritance of the gRNA HEG element as germline expression of the nuclease was. However, in these first crosses the gRNA gene was contributed by the grandfather, providing no opportunity for the Cas9:gRNA to complex before being deposited. In a subsequent cross with the grandmother carrying both the Cas9 and gRNA genes, the estimated homing rate dropped sharply to 9.2% in trans-heterozygotes (gRNA + Cas9) and to 6% in heterozygotes (gRNA only). When both Cas9 and gRNA were maternally deposited, cleavage could occur in the early embryo, forming resistance alleles, which then prevented drive conversion at a more opportune stage. This did not occur with maternal deposition of only Cas9 (not gRNA) into an individual that can nonetheless express the gRNA. gRNA expression, thought to be constitutive, and subsequent complex formation with the deposited Cas9 protein seemingly limited cutting to a more opportune stage for inheritance bias even in the absence of Cas9 expression. A similar result was reported by López Del Amo et al., with Cas9 and gRNA carrying mothers having a detrimental effect on inheritance bias by their progeny while contribution of the gRNA from the father and Cas9 from the mother did not (López Del Amo et al., 2020a).

## 4 What Strategies do we Have to Combat These Challenges?

### 4.1 Achieving Restricted Nuclease Expression

Restricting nuclease activity to cells and developmental stages where HDR is expected to be favoured and somatic tissues are unaffected is an optimisation strategy commonly pursued within the field. This is especially important in population suppression drive systems which rely heavily on the fitness of drive-carrying heterozygous (female) individuals (Eckhoff et al., 2017; Beaghton et al., 2019; North et al., 2020; Champer et al., 2021a,b). To achieve this restricted activity, the field has largely relied on identifying and testing multiple genes which are predicted to have the desired expression/activity profile. The putative regulatory sequences of these genes are then isolated and used to express Cas9. This strategy has shown success such as the improvements achieved by *zpg* expressed Cas9 in *A. gambiae* compared to Cas9 expressed with *vasa*2, *nanos*, or *exu* (Hammond et al., 2021a). This change of expression resulted in the reduction of both somatic drive activity and deposition of the nuclease while maintaining the inheritance biasing efficiency. While this trial-and-error approach has yielded improved HEGs in multiple species, insights into what underlies any improvement in performance are very limited as many changes are made simultaneously that cannot be deconvoluted.

The future design of HEGs may be aided by studying the effect of “stacking” multiple limited regulatory mechanisms. In addition to the use of promoter/5′UTR and 3′UTR sequences, other endogenous regulation mechanisms can be included, such as tissue-specific splicing (Salles et al., 2002; Tsujimoto et al., 2013; Sutton et al., 2016), modulation of protein degradation (Chassin et al., 2019), sub-cellular localisation (Goeckel et al., 2019), and inclusion of miRNA binding sites (Loya et al., 2009). Ideally each regulatory system should make as limited and well defined a change as possible. Decoupling of expression timing from expression levels may be a useful first candidate as the stacking of regulatory mechanisms may be expected to cause a cumulative decrease in activity levels due to imperfect removal of inhibition. Grunwald et al. demonstrated this principle with a HEG by using a strong and constitutive promoter to express Cas9 that could only be translated once a stop codon had been excised by separate and germline-restricted expression of a recombinase (Grunwald et al., 2019). An important downside of Grunwald’s approach was that once activated, the Cas9 could no longer be shut-off. A similar approach, but using a transcription factor intermediate such as the GAL4–UAS system would allow for reversible activation (Fischer et al., 1988). Such a system may particularly benefit “integral” gene drives.

Integral gene drives make direct use of an endogenous gene for their expression (Nash et al., 2019). Hoermann et al. demonstrated, in three different genomic loci of *A. gambiae*, that an artificial intron can be used to express both an effector protein and the host gene product (Hoermann et al., 2021). A gRNA targeting the unmodified locus was also included, which allowed for efficient inheritance bias of the whole element with a separately expressed Cas9. Inclusion of the large Cas9 gene ( > 4 Kb) in an integral gene drive context has been recently demonstrated in mice, where Cas9 was integrated at the very end of the coding region of the *Spo11* gene (Weitzel et al., 2021). This design resulted in Cas9 being co-translated with the endogenous gene, with the aim of restricting Cas9’s activity to match that of *Spo11* which is involved in meiotic recombination. Using this approach, Weitzel et al. demonstrated for the first time homing in male mice despite earlier efforts with other regulatory systems (Grunwald et al., 2019; Weitzel et al., 2021). However, the majority of target alleles remained uncut, indicating that improvements in regulating the timing of Cas9 activity came at the cost of its activity level. An intermediate amplifier of expression may address these expression issues and, if smaller than Cas9, interfere less with the endogenous gene’s function (the endogenous *Spo11* gene was impaired by the Cas9 insertion).

Finally, there may be specific interventions that can address the effect of Cas9 deposition. In a study in *A. gambiae*, the I-*Ppo*I homing endonuclease was expressed by the testis-specific promoter *β* *-tubulin* to establish a synthetic sex ratio distortion system by shredding the X chromosome in the paternal germline (Windbichler et al., 2008). However, no viable embryos were produced because paternally deposited I-*Ppo*I also shredded the maternally contributed X chromosome in the zygote. A subsequent study was carried out to reduce the half-life of the endonuclease by systematically introducing point mutations into the protein (Galizi et al., 2014). Strains with high levels (95–97.4%) of male-biased sex distortion and fertility rates similar to controls were eventually generated using this approach. Interestingly, the modified I-*Ppo*I was recently combined with a HEG drive system into a “sex-distorter gene drive” (Simoni et al., 2020). When tested at three new loci, expressed with the identical *β* *-tubulin* promoter, male sterility was reestablished to varying degrees, presumably due to locus-dependent changes in expression of the I-*Ppo*I endonuclease, causing sufficient protein to persist into the embryo. By introducing a 100-bp GC-rich DNA sequences into positions -271, -244, and -355 upstream of the start codon, respectively, transcriptional activity of the *β* *-tubulin* promoter was reduced to 0.5, 8.1, and 16.2%. The promoter variant with 8.1% transcriptional activity, coupled with a destabilised I-*Ppo*I, was inserted into the *dsx* locus and was found to have no detectable sterility in drive heterozygous males. Similar approaches may also address deposition of Cas9 in a HEG context.

### 4.2 Multiplexing

The targeting of multiple sequences (“multiplexing”) has been proposed as a means of addressing one of the most significant impediments to HEG drives - resistance (Esvelt et al., 2014). If an initial attempt at homing fails and induces a mutation, multiplexing may still allow for homing through cleavage at an alternate cut site. Moreover, multiplexing would also allow the HEG to drive in individuals that have preexisting sequence variation in a subset of cut sites. Another benefit, specifically when targeting high-fitness cost genes, is that for complete resistance (resistance at all target sites) more extensive sequence changes would need to occur and this reduces the likelihood of the formation of type-1 resistance mutations. In terms of feasibility, multiplexing is particularly convenient with CRISPR-Cas9 nuclease as it only requires expressing additional gRNAs. Under these assumptions, computational modelling has indicated multiplexing to be an effective strategy to reduce the formation and accumulation of resistant alleles (Marshall et al., 2017; Noble et al., 2017; Prowse et al., 2017; Champer S. E. et al., 2020; Edgington et al., 2020). However, some practical challenges have emerged with this “classical” multiplexing approach.

**FIGURE 5:**
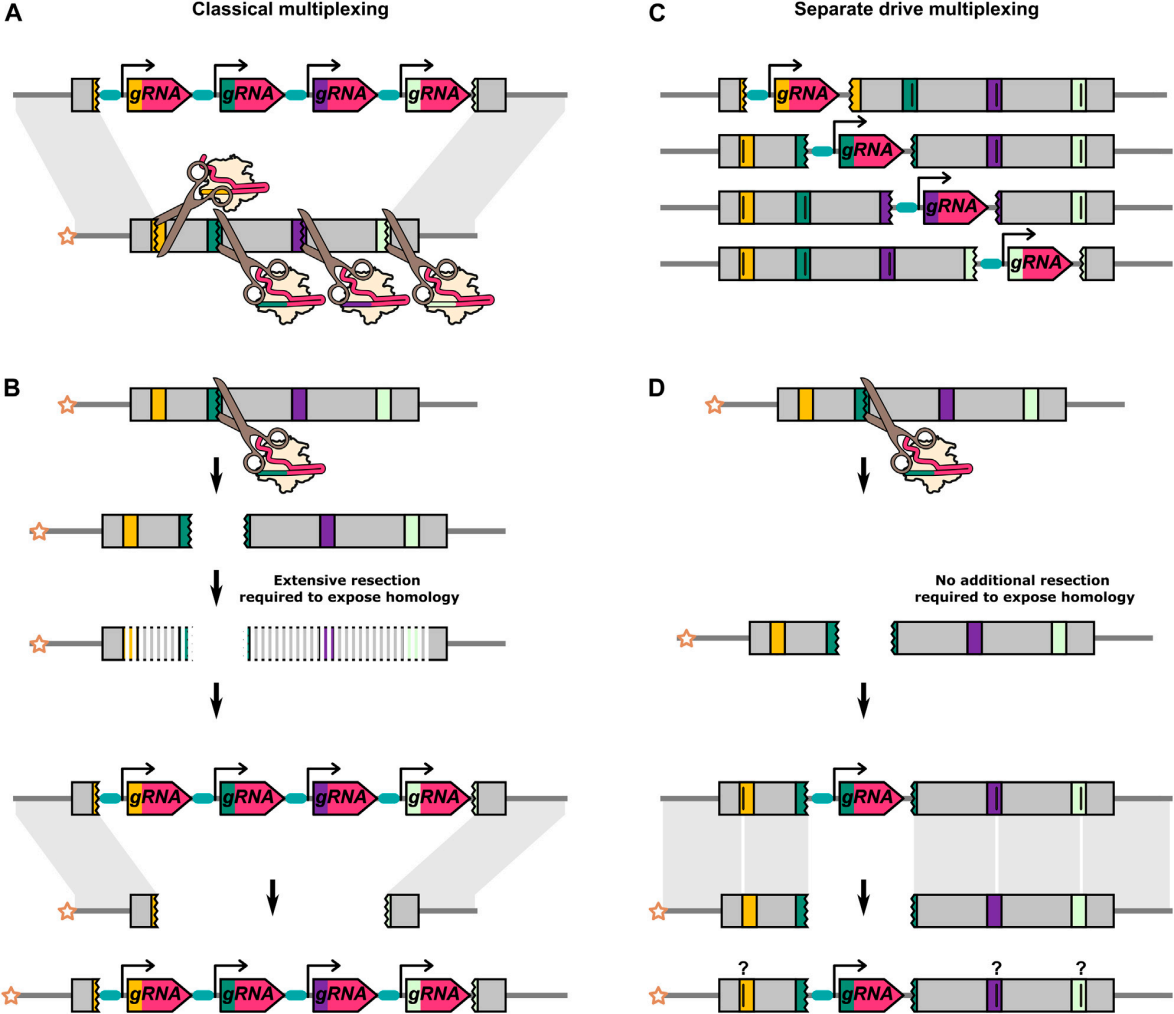
Differences between classical multiplexing and separate drive multiplexing. **(A)** Classical multiplexing construct targeting adjacent sequences within a single gene. Four gRNAs are encoded from a single construct. **(B)** When the outermost gRNAs do not cleave the recipient chromosome simultaneously, the cut caused by any one of the gRNAs will inevitably result in a region of extraneous sequence on the recipient chromosome which is “unmatched” to the donor chromosome. As further 5′-3′ and 3′-5′ resections have to occur prior to HDR, this might reduce HDR efficiency and favour NHEJ. **(C)** Four separate drive elements targeting adjacent sequences within a single gene. These are independent modifications of the same target gene and are not present in the same individual. Alternatively, separate drive constructs could be used to target multiple loci at distinct sites within the genome. **(D)** The cut caused by the gRNA on the recipient chromosome is repaired by using the “matched” homology arm on the donor chromosome as its template. The separate drive elements can include recoded target sequences for the other gRNAs to prevent cutting between elements. It is unclear if and how these types of sequence changes would affect homing efficiency. In A, one gRNA target is shown to be cut in the opposite orientation to the others. The different orientation is to do with the asymmetrical position of the cut site within the standard 20bp gRNA binding site (17bp//3bp). After a cut with a single gRNA, one end of DNA break will have at least 17bp of homology with the gRNA gene, and the other end will have at least 3bp. With a multiplex design, the outermost gRNAs can be oriented opposite to each other such that both DNA ends carry either the smaller or the larger region of homolgy to the gRNA gene. A DNA end with only 3bp of homology to the gRNA gene will presumably minimise the risk of partial homing/internal recombination. In contrast, if resection after a single cut (see B) is to be minimised, the opposite gRNA orientation may be desirable. For gRNA target sites that are not in the outermost position, there may be no overall “optimal” orientation as it varies with the particular combination of gRNA targets that have cut.

In the classical approach to multiplexing, multiple gRNAs targeting closely linked adjacent sequences of a single gene are expressed in a single drive transgene (Figure 5A). Additional sequences need to be removed or replaced on the donor chromosome to prevent the HEG from cutting itself at these additional sites. For a single target HEG, the ends of the cut site can be perfectly homologous to the donor chromosome. However, in a multiplexing system, any individual cut site can no longer generate two DNA strands that are perfectly homologous to the donor chromosome (Figure 5B). There are indications that these extraneous, “unmatched” sequences could reduce the homing efficiency (Champer et al., 2018, Champer et al., 2020 S. E.; López Del Amo et al., 2020a), presumably due to the additional resection that would not need to occur prior to HDR with a perfectly homologous repair template (Liu and Kong, 2021; Ang et al., 2022). López Del Amo et al. introduced 20bp truncations in the homology arms either side of a *D. melanogaster* HEG (López Del Amo et al., 2020a). These truncations result in 20bp of unpaired sequences on the recipient chromosome that would normally be homologous to the sequence directly adjacent to the HEG. The inheritance biasing rate of the HEG was significantly reduced with truncation on both sides of the HEG. Consistent with this, two HEGs, each with four gRNAs, targeting sites spread over a large region ( > 2 Kb) (Oberhofer et al., 2018) performed worse than similar drives with one gRNA (Champer et al., 2017) or two gRNAs targeting a smaller region (Champer et al., 2018). Moreover, additional gRNAs may compete to complex with a limited amount of Cas9 protein lowering the cut rate at any one site (Champer S. E. et al., 2020). These results indicate that any individual cut site in a classical multiplexing may be less efficient than a drive element optimised for only one cut site. Nonetheless, additional studies have indicated that multiplexing can increase the overall efficiency of a HEG albeit in some cases with diminishing returns for additional gRNAs (Champer et al., 2018, Champer et al., 2020 S. E.; Yang et al., 2021), and (López Del Amo et al., 2021) compared to (López Del Amo et al., 2020b).

A novel challenge introduced by multiplexing is expressing different gRNAs simultaneously at similar concentrations without introducing repetitive sequences. Multiple strategies have been proposed to achieve this, many of which involve the excising of individual gRNA from a single long transcript such as with tRNAs (Port and Bullock, 2016; Knapp et al., 2019). However, these excising approaches are frequently not perfectly efficient or leave scars in the form of additional nucleotides attached to the gRNA that can reduce their activity. A separate approach that may be effective is for each gRNA using a set of different (minimal) promoters that have been characterised to have similar expression levels (Anderson et al., 2020). Using different experimentally validated “backbones” for the non-targeting sequences of the gRNA may further reduce the likelihood of internal recombination (Noble et al., 2019).

Cas9 is known to remain bound to its target even after making a double-strand break (Sternberg et al., 2014), and DNA repair occurs more slowly than with other sources of DNA damage (Brinkman et al., 2018). This may provide an opportunity for DNA breaks to occur at different sites before DNA repair is completed, leading to frequent deletions between independent target sites (Brinkman et al., 2018), as has been observed for with some multiplexing HEGs (Champer et al., 2018; Oberhofer et al., 2018; Champer S. E. et al., 2020). In addition, CRISPR-Cas9 has been found to frequently induce large deletions ( > 250) at single cut sites (Adikusuma et al., 2018; Kosicki et al., 2018; Nambiar et al., 2022). Large deletions, or simultaneous cutting of at least the outermost target sites of a multiplex drive, could remove all unmatched sequences from a HEG recipient chromosome, potentially restoring homing efficiency to the level of a single target drive. However, this would also negate some of the benefits of multiplexing, as it may cause the simultaneous loss of all gRNA recognition sites on the chromosome. Recently, a separate drive or multi-locus multiplex strategy has been proposed that may avoid some of the diminishing returns of classical multiplexing.

Multi-locus multiplexing consists of multiple ‘parallel’ single-target HEGs generated as separate lines, each targeting an adjacent site in the target gene (Figures 5C,D) (Edgington et al., 2020). Compared to the classical multiplexing strategy, the separate drive approach will be logistically more onerous but benefits from not being able to generate deletions of all target sites by simultaneous cutting. However, experimental validation of this approach has yet to be reported.

### 4.3 Targeting Essential Genes

The targeting of essential genes with HEGs has been reported in many studies, but depending on the goal of the system (i.e, suppression or modification), the approaches may diverge. For a suppression drive, the goal is generally to disrupt the essential gene in individuals of the target population. To achieve this, a functionally constrained sequence can be targeted such that few, if any, cut-resistant mutations generated will have a fitness advantage over the drive allele. In *A. gambiae*, an ultra-conserved region of a female-specific isoform of the *doublesex* gene was targeted to impede formation of resistant alleles (Kyrou et al., 2018). HEG drives based on this target site in three cage trials were able to cause complete population crash, and no type-1 resistance alleles were recovered (Kyrou et al., 2018; Simoni et al., 2020; Hammond et al., 2021b). A similar drive, targeting an ultra-conserved exon in a different gene did lead to the emergence of resistance to the HEG in the form of a single nucleotide silent mutation (Fuchs et al., 2021). The target site to which no resistance emerged was located at an intron-exon junction, potentially making mutations liable to disrupt crucial mRNA secondary structures.

**FIGURE 6:**
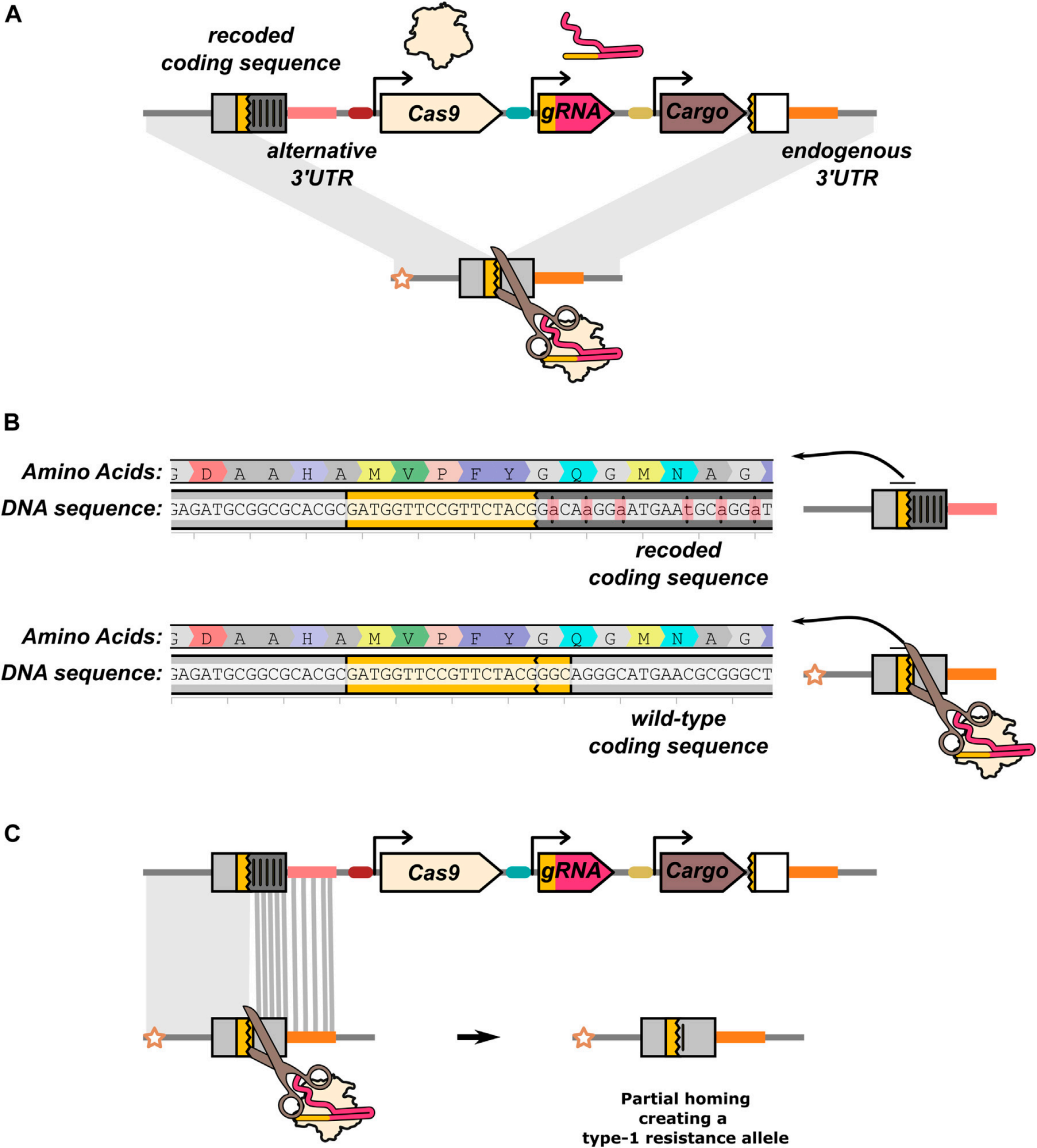
Rescue HEG targeting an essential gene. **(A)** In addition to the nuclease components (Cas9 and gRNA), the drive element carries an effector/cargo gene and a “rescue” sequence that restores the function of the gene the HEG is disrupting. The rescue sequence allows the drive element to be more fit than type-2 resistance alleles. The effector function of the rescue HEG is mediated by a separate cargo gene. In all other illustrations in this manuscript, the effector function of the drive is assumed to be mediated by disruption of the target gene and the HEG does not carry a rescue. **(B)** In this example, the rescue is a recoded version of an endogenous gene. Synonymous codon changes have been made to prevent partial homing (and recognition by the gRNA). **(C)** The recoded gRNA target sequence is located at the start of the rescue sequence. Partial homing by means of any part of the recoded sequence would generate a type-1 resistance allele. The recoding and gRNA target sequence is taken from (Pham et al., 2019).

Targeting an essential gene can also be used for non-suppression drives as a general approach for lowering the viability of mutations (“home-and-rescue”/rescue HEG). By providing a rescue sequence within the HEG drive construct (Figure 6A), resistance can be mitigated as the HEG drive will now have a fitness advantage over type-2 resistance alleles. The role of the rescue sequence is well illustrated by the first HEG drive developed in *A. stephensi*. This drive was inserted into an eye pigmentation gene, *kmo* (Gantz et al., 2015), which was later found to have a recessive fitness cost in females (Pham et al., 2019). This reduced fitness, coupled with a reduction in inheritance biasing efficiency in individuals experiencing maternal deposition caused the drive to fail to reach fixation when released at a 1:10 drive:wild-type ratio and even performed poorly at a 1:1 ratio (Pham et al., 2019). A new version of the HEG was developed that included recoded parts of the *kmo* gene resulting in the drive allele no longer disrupting its function. A subsequent cage trial demonstrated this improved version of the HEG could effectively spread and reach fixation (Adolfi et al., 2020). In addition to biasing its inheritance by cutting target alleles, the improved HEG could also increase in frequency by positive selection when the frequency of type-2 alleles accumulated in the population.

The most common approach for rescuing the function of the target gene is providing a “recoded” version of the sequence the drive allele is disrupting (Figure 6B). Like type-1 resistance alleles, these are sequence changes (such as the swapping of synonymous codons) that prevent recognition by the gRNA but leave the target gene functionally intact. While in some cases a single nucleotide substitution can be sufficient to prevent Cas9:gRNA binding, much more extensive recoding is performed to reduce the risk of partial homing (Figure 6C). Recoding is further complicated by the need to include noncoding sequences such as the 3′UTR, for which no straightforward synonymous sequence substitution rules exist. In the case of the *kmo* targeting *A. stephensi* drive, the 3′UTR from the *A. gambiae kmo* gene was used in the rescue element (Adolfi et al., 2020).

The manner in which positive selection is conferred to the recoded rescue allele is similar to that expected in a Cas9-based toxin-antidote system where Cas9-induced mutations cause lethality or sterility allowing a cut-resistant rescue/antidote gene to spread in the population (Oberhofer et al., 2019; Champer et al., 2020a). Moreover, deposition can increase the effective inheritance rate of the drive by culling individuals that have inherited a type-2 allele from their drive carrying mother and a functional target allele from their father. Disruption of the paternally contributed target allele by maternally deposited nuclease can make these individuals no-longer viable. Progeny that inherited the drive allele will be protected as they carry the (dominant acting) recoded antidote. However, these drive inheriting individuals will likely have severely reduced homing rates. These rescue HEG systems have been reported in several studies targeting haplolethal (*RpL35A* (Champer et al., 2020b)) or haplosufficient (*rab5*, *rab11*, *spo11*, *prosalpha2*, and *PolG2* (Terradas et al., 2021b; Kandul et al., 2021)) genes. All three of these studies showed increased inheritance of the rescue alleles to varying degrees and demonstrated this strategy to be successful in mitigating the negative effects of type-2 alleles to the drive system.

In the study by Champer et al., multiplexing (two gRNAs) was combined with targeting the haplolethal *RpL35A* gene (Champer et al., 2020b). When the target gene is haplolethal instead of haplosufficent, a single rescue gene may not be sufficient to protect from the lethal effects of deposition. This makes deposition a much more substantial hurdle for these types of systems. Shadow drive may theoretically be expected to create viable progeny by homing an inherited rescue element or a type-1 resistance allele. However, rescue through shadow drive may be very unlikely as deposition often results in mosaic outcomes. Depending on the target gene, any individual progeny may have a significant proportion of cells that have not been rescued by shadow drive and therefore nonetheless become inviable (“lethal mosaicism”). Similarly, with a haplosufficient target, individuals inheriting a maternally contributed type-2 resistance allele may not be rescued by mosaic type-1 alleles produced by deposition-induced cutting.

## 5 Concluding Remarks

Synthetic homing endonuclease gene drives have been actively researched for over a decade. In this time, the field has developed and characterised a range of designs and applied these to a diverse set of species. Insects, and specifically *Drosophila* and the *Anopheles* mosquitoes, have so far proved substantially more amenable to HEG mediated inheritance bias than other animals and plants. Additional work will prove if the optimisation approaches developed in these insects can be successfully applied in these other species. This effort may be aided by a more systematic and high-throughput HEG test and design approach.

While efforts have been made to experimentally validate the intended transgene expression pattern (Hammond et al., 2016; Kandul et al., 2020; Terradas et al., 2021c; Weitzel et al., 2021), there are fewer cases where this has been done throughout development (Li et al., 2017). The result of this is that any hypothesis about the ideal expression/activity pattern for HEGs is currently essentially unfalsifiable as any exception to a proposed hypothesis can easily be explained away by the many ways a drive may fail to recapitulate the intended expression pattern, at the needed expression level. Improved methods to test different activity patterns (e.g., drug inducible Cas9 (López Del Amo et al., 2020b; Chae et al., 2020)) and high-throughput methods to track HEG expression and nuclease activity (e.g., Cas9-based lineage tracing (McKenna et al., 2016)) are sorely needed to validate our assumptions about the underlying factors influencing interchromosomal HDR. This becomes increasingly important as evidence emerges that interchromosomal HDR can occur before the formation of the mature germline (López Del Amo et al., 2020a; Kandul et al., 2020; Filler-Hayut et al., 2021; Li et al., 2021).

Many approaches have been developed to control the expression and activity of transgenes. However, the use of modular systems such as GAL4–UAS to enforce a new HEG activity pattern will likely be more challenging than the current trial-and-error approach of identifying and testing new promoter/5′UTR and 3′UTR regulatory sequences. Yet, we expect that this type of modular approach will enable high-throughput design-build-test cycles. The modularity gained with such an approach will in turn improve our ability to draw conclusions about the underlying biology affecting HEG efficiency and increase the robustness of new designs going forward.

While multiplexing may have diminished returns in improving homing efficiency with standard approaches, it may nonetheless greatly diminish the likelihood of type-1 resistance alleles emerging. The targeting of highly conserved sequences in essential genes has proved beneficial for reducing the impact of resistance alleles. If approved, we expect that the current “state-of-the-art” HEGs in *Anopheles* mosquitoes may progress on to field trails without substantial additional changes to their core design. If this is the case, the complexity of a real-world release will be the ultimate test of the HEG technology. Our constantly expanding genetic “tool-box” and optimisation strategies provide hope that HEGs may be a highly effective tool for combating the harms caused by a broader set of medically and agriculturally relevant (homing refractory) insect pests.

## Glossary

**Gene drive:** Gene drive refers to both the process by which a genetic element biases its own inheritance, and the genetic element itself (Alphey et al., 2020). Frequently shortened to just “drive” for either meaning.

**Homing endonuclease gene (HEG) drive:** A gene drive can be composed of multiple distinct genetic elements. A form of gene drive that functions through the cleaving of a specific genomic locus followed by homology-directed repair (HDR) using the homologous chromosome as the template. Inheritance of the drive allele is increased when the nondrive allele is converted to a drive allele in the germline linage of an individual heterozygous for the drive. In the context of this review, different implementations of the core HEG design will be discussed as different drives. HEG-based drives are also commonly referred to as “homing drives”.

**Nuclease:** An enzyme that can catalyse the cleavage of nucleic acids. For HEGs, sequence-specific endonucleases are used.

**CRISPR-Cas9:** Clustered Regularly Interspaced Short Palindromic Repeats Associated Protein 9. An endonuclease protein that can be programmed to specifically cut a DNA sequence through a guide RNA (gRNA). Cleavage requires an additional three base-pair protospacer adjacent motif (“NGG”) specified by interactions of the Cas9 protein with the target DNA.

**gRNA:** A ≈100 nucleotide RNA that complexes with the Cas9 protein and specifies a ≈20 nucleotide long target sequence to be cut by RNA:DNA base-pairing.

**Gene drive allele/element:** Genetic components that contain all or a subset of the genetic parts needed to induce gene drive. Gene drive mechanisms rely on endogenous pathways (such as DNA repair), but these are not considered part of the gene drive itself.

**Target allele:** The target, often wild-type, allele that the HEG can cut and convert. It can also be a “synthetic” target site specifically introduced to contain the HEG’s recognition sequence. In research contexts, the target is often a (haplosufficient) gene that, when disrupted, gives a phenotypic readout.

**Donor and Recipient chromosome:** In a heterozygous individual, the HEG is designed to copy itself from the “donor” chromosome to the “recipient” chromosome. In some studies, the homologous chromosomes can be differentiated by a marker separate from the drive allele. This allows the original donor alleles to be distinguished from the newly created drive alleles on the recipient chromosome.

**Homing/Drive conversion rate:** Conversion of a target allele to a drive allele through interchromosomal HDR. Commonly measured as the fraction of target alleles that have been converted to a drive allele in the germline lineage of a drive allele heterozygote. If either the target or the drive allele have a tightly linked marker, this can be calculated very precisely. If not, it is estimated from the inheritance of the drive compensated for the 50% expected inheritance from a heterozygote through nondrive Mendelian inheritance.

**Type-1 resistance mutations:** A functional classification of mutations in the HEG target gene that leave the target gene’s function intact. This is target dependent, and the same sequence changes can result in different functional outcomes for different genes and different sites within the same gene.

**Type-2 resistance mutations:** Mutations in the HEG target that disrupt the gene’s normal function.

**Unconverted target alleles:** Target alleles that have not been mutated or converted by the HEG. It is possible that the unconverted target alleles were cut, but DNA repair did not introduce any mutations. For many crosses, without additional molecular assays, intact target alleles cannot be phenotypically differentiated from type-1 resistance mutations.

**Deposition:** HEG nuclease protein, mRNA, or gRNA that has been expressed in the parent and carried over to the progeny. Deposition does not require the progeny to have inherited the gene that expressed those HEG components in their parent(s). However, the inheritance of a HEG allele can influence the outcome of deposition.

**Shadow drive:** Biasing of a particular allele, presumably through homing, that occurs as a result of deposited HEG components.

**Somatic phenotype:** HEGs are commonly tested by targeting a gene that gives a somatic phenotype when disrupted, such as a yellow body or white eyes in insects. For many experimentally tested HEGs, heterozygous individuals do not present with a somatic phenotype unless nuclease activity has caused the wild-type target allele to be replaced by the drive allele or a type-2 resistance mutation in a substantial proportion of somatic cells.

**Knock-out phenotype:** Uniform phenotype which is consistent with the target gene having been disrupted in most or all cells of that tissue (e.g., completely white eyes).

**Mosaic phenotype:** An intermediate phenotype between wild-type and the knock-out phenotype consistent with some cells within the organism retaining function of the target gene and the target gene having been disrupted in other cells.

**Somatic nuclease/drive activity:** Activity of the HEG nuclease in somatic tissues. In almost all cases this is not intended.

**Germline restricted nuclease/drive activity:** Activity of the HEG nuclease in cells of the germline. Characterised by changes to the gamete frequencies without generating a somatic phenotype.

**Embryonic nuclease/drive activity:** Activity of the drive in the early embryo. Often characterised by both (error-prone) germline and somatic drive activity. Associated with individuals that have experienced parental deposition.
